# On Catching Cold

**Published:** 1875-03

**Authors:** 


On Catching Cold.—This abstract is part of a popular lecture recently delivered in London by Dr. E. Symes Thompson, clipped from the *Evening Post.* Physicians have much to answer for from their neglect to explain in familiar ways the principles underlying the etiology of ordinary catarrhs. If more stress were laid on the risk of going into overheated rooms, instead of coming out of them, people would become wiser and healthier. Nine-tenths of the ordinary annoying colds are taken in that way.

"In regard to prevention, he deprecated too much fear of catching cold and the dread of the least exposure to cold air, as being very likely to bring about that tender hothouse-plant condition so favorable to inducing the evil one wished to avoid. Exposure to cold air, he insisted, does no harm, except under the condition of its moving rapidly in a small space. Thus exposure to strong wind in the open air does no harm, while a Portugese proverb said with a great deal of truth, if you catch cold from a draught through a keyhole you had better make your will. It was draughts coming with great rapidity through small openings which were so especially injurious. Living constantly in very impure air made people very sensitive to cold, and ill-ventilated bed-rooms had much to answer for in this respect. It was a mistake to suppose that night air, *except in aguish places*, was obnoxious, and every one, while avoiding a direct draught, should keep the bed-room window slightly opened. In clothing, vary the character and amount according to the season, avoiding the extremes of always being swathed in flannel no matter what the temperature; or of never wearing flannel at all. Of the curative treatment the "dry method" had once been in great vogue. This consisted of abstaining from all fluids for twenty-four, thirty-six, or forty-eight hours, and where rigorously followed at the outset the cold was generally stopped. He would not recommend this treatment to any but those in thoroughly good health, for in the delicate or the sickly the derangement of the vital organs, especially the liver and the digestive organs, by this abstention from fluids, brought about evils more serious than the cold. Another method was the maintenance of an equable warm temperature, and where this could be done the skin was soon restored to a more natural condition, and the evil was relieved. The mucous lining, however, could be more rapidly relieved by inducing the skin to perspire vigorously, and if this was done at the outset the cold would be checked. This could be done by a hot bath; or, very much better, by a Turkish bath, for while in a hot-water bath it was not possible to endure a greater heat than 100 to 103 degrees, in a Turkish bath a temperature of 150 to 200 degrees could be sustained without discomfort. Vigorous perspiration was thus induced, the blood was drawn from the internal organs to the surface, much of its impurity eliminated, and if the cold douche was avoided and the skin was got thoroughly to work, the patient walked away in an hour, well."

The writer once relieved a violent coryza in his own case in this way. On rising he swallowed a full saline cathartic, inducing watery stools. During the day he reduced the fluids ingested to the minimum. In the afternoon he took an ordinary hot bath, keeping the temperature up to the highest endurable point and remaining in the water to the verge of faintness. Vigorously rubbing himself dry completed the cure.

